# Empirical Study and Improvement on Deep Transfer Learning for Human Activity Recognition

**DOI:** 10.3390/s19010057

**Published:** 2018-12-24

**Authors:** Renjie Ding, Xue Li, Lanshun Nie, Jiazhen Li, Xiandong Si, Dianhui Chu, Guozhong Liu, Dechen Zhan

**Affiliations:** School of Computer Science and Technology, Harbin Institute of Technology, Harbin 150001, China; renjie_ding_hitwh@163.com (R.D.); lixuecs@hit.edu.cn (X.L.); lijiazhen_smile@163.com (J.L.); 15776633420@163.com (X.S.); chudh@hit.edu.cn (D.C.); liuguozhonghit@163.com (G.L.); dechen@hit.edu.cn (D.Z.)

**Keywords:** human activity recognition, transfer learning, deep learning, sensor data

## Abstract

Human activity recognition (HAR) based on sensor data is a significant problem in pervasive computing. In recent years, deep learning has become the dominating approach in this field, due to its high accuracy. However, it is difficult to make accurate identification for the activities of one individual using a model trained on data from other users. The decline on the accuracy of recognition restricts activity recognition in practice. At present, there is little research on the transferring of deep learning model in this field. This is the first time as we known, an empirical study was carried out on deep transfer learning between users with unlabeled data of target. We compared several widely-used algorithms and found that Maximum Mean Discrepancy (MMD) method is most suitable for HAR. We studied the distribution of features generated from sensor data. We improved the existing method from the aspect of features distribution with center loss and get better results. The observations and insights in this study have deepened the understanding of transfer learning in the activity recognition field and provided guidance for further research.

## 1. Introduction

Human activity recognition has received considerable attention in the field of pervasive computing. With the widespread use of electronic devices, human activity recognition based on sensor data has become a major research trend. The purpose of this research is to recognize the activities of the human body with the data collected by sensors. Deep learning is becoming the mainstream approach due to its high accuracy [1], such as Convolutional Neural Networks (CNN) [2], Long Short-Term Memory (LSTM) [3] and so on.

**Figure 1:**
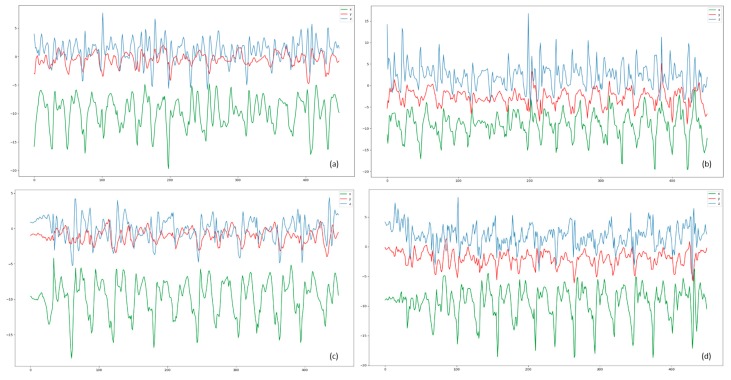
The curve of data from acceleration. (**a**) User A downstairs. (**b**) User B downstairs. (**c**) User A upstairs. (**d**) User B upstairs.

When using sensor data to train a model, the accuracy is generally high if the training set and test set belong to the same collection of users. When it comes to different user sets, the accuracy rate will drop obviously. There are several reasons why transfer learning is necessary in HAR. (1) When users perform the same activity, the data collected by sensors tends to be quite different because of the differences in physiology and habits (Figure 1). (2) The movement of user will change over time as well. The characteristics of the teenagers and the elderly are very different as well. (3) If deep learning wants to be applied for HAR in practice, it will be faced with large number of emerging users. It is impractical to train a model for each user with collecting large labelled data. (4) Collection and labeling of data for HAR is really time consuming, especially for the elderly.

The cost of collecting some unlabeled data for new users is more acceptable. Thus, we want to carry out the unsupervised transfer learning. The unsupervised transfer learning means source domain has labeled data and target domain has unlabeled data here. There are some works about transfer learning in HAR. They transfer between users, body parts, locations, devices and so on. Most of them used machine learning methods [4,5,6]. For deep learning, the researchers used some labeled data in target domain to fine-tune the model [7]. What we want to do in this work is to transfer the deep learning model between users with unlabeled data of target.

We investigated the problem here. Initially, the data collected from existing users is labeled. We defined the existing user as a source domain: $D_{S}=\left\{x_{s},\;y_{s}\right\}$. Since our target user is often new to the study, it is difficult to collect large amounts of labeled data, so we assume that the only data available is unlabeled. We define the target user as a target domain: $D_{t}=\left\{x_{t}\right\}$. Our purpose is transferring a deep learning model derived from $D_{S}$ to $D_{t}$, in order to obtain a suitable HAR model for the target. 

The main contributions of this paper are as follows:

(1) For the first time in HAR, a large number of empirical studies have been conducted on unsupervised deep transfer learning methods. We compared several widely-used methods and found the MMD method is most suitable. Some useful insights were generated as well.
(2) The feature distribution of sensor data extracted by CNN was analyzed. We found that the inner-class distance (the distance of each feature to the center of its own class) is large, and the inter-class distance (the distance between centers from different classes) is small. These two distances affect the transferring.
(3) In order to verify that improving feature distribution is helpful for unsupervised transferring, we combined the MMD method with Center Loss. The inner-class distance can be decreased by Center Loss, thus the features from different classes will spread out relatively. The sharpening of the source classification is helpful for transferring.

## 2. Related Work

### 2.1. Human Activity Recognition

Many studies focus on the recognition of daily and sports activities. Deep learning is being used more and more widely. Some researchers modified the convolution kernel to adapt to the characteristics of triaxial accelerometer data and built CNN network for training and learning [2]. Unlike images only have spatial connection between pixels, sensor data is a time series as well. Therefore, the models for time series are widely used in HAR. LSTM is used to predict human activities with the data collected by sensors of a mobile phone [3]. Edel et al. [8] solved the problem of activity recognition with Bi-directional LSTM (Bi-LSTM). There is also study which extracted both space and time features [9]. It combined four layers of CNN with two layers of LSTM and reached better results than only CNN.

Activity recognition is currently used in many fields. In the medical field, researchers attached wearable sensors to shoes to reflect the speed and gait of people walking [10]. They used this data to identify individuals with Parkinson’s disease. Recognition and monitoring of activity for sports is also an important area. Single person movement [11,12] and multiplayer confrontation [13] have been studied. Researchers have collected data from athletes with wearable devices to study and predict their shooting actions [11]. Miikka et al. [12] found that the accuracy decreased by 17% unit when only supervised data were used for training and only unsupervised data for validation. In the future, we expect more research in sports training, behavior prediction and other related areas.

### 2.2. Transfer Learning

Transfer learning has achieved successful applications in many fields, such as computer vision, NLP. In one image classification problem, researchers transferred models between different image data sets to make the classifier adaptive [14]. Some researchers studied the switching of conversation models in different scenarios within NLP (Natural Language Processing), so that the dialogue system can satisfy the needs of users [15].

We focus on the transfer learning based on features. Previous work has demonstrated the use of transformation to map features from the source and the target into the same high-dimensional space, enabling the algorithm to use features in the same space to train the classifier [16,17]. In paper [18], they proposed the method using MMD to measure the difference between source and target. These authors find a way to minimize this discrepancy to transfer. There are several excellent methods based on MMD and deep learning, such as Deep Domain Confusion (DDC) [19], Deep Adaptation Networks (DAN) [20]. 

Some other work has involved instance-based transfer learning methods. These methods [21,22,23] used samples of source to train the model, then they picked out samples from source which are most similar to ones in target, then they use these samples to fine-tune the model. Other methods are transferring based on relationships [24,25], they study the relationships of network trained on source data and made use of these information in the target domain. Finally, some methods [26,27] make use of pre-trained model which is trained on source data. They keep the parameters of the model and use slide patches of target to fine-tune the new adaptation layers.

### 2.3. HAR Based on Transfer Learning

At present, the transfer learning methods used in HAR are mostly based on machine learning. They [4] combined naive Bayes with Support Vector Machine (SVM) and the accuracy increased by 6% for new users. Paper [5] proposed a method combining the expectation maximization (EM) algorithm and the conditional random field (CRF) algorithm to transfer between different data sets with physiological data. Zhao et al. [6] used decision tree with k-means and iterate with fake labels to improve the accuracy for new users. 

Paper [7] first explored the deep transfer learning within HAR, these authors used three layers of CNN with one layer of LSTM, fine-tuned the model with freezing different layers to study the transferring between users, locations and devices. Wang et al. [28] proposed the Unsupervised Source Selection algorithm for Activity Recognition (USSAR) method to study the transferring among different body parts of human.

## 3. Empirical Study on Unsupervised Transfer Learning Methods

### 3.1. Methods We Used

#### 3.1.1. Basic Deep Learning Model

**Figure 2:**
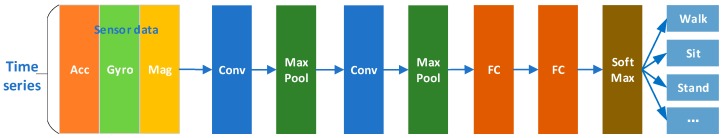
CNN framework we used. We used two convolutional layers, two pooling layers, two fully connected layers. The last fully connected layer and Softmax form a classifier.

We used a CNN as the base model, which is shown in Figure 2. We used data collected by several sensors as single-channel images. We used this approach to learn from the source data, and then recognize target data directly. This is called as an only-source approach.

We used this CNN as the basic model, because the Transfer Learning methods used below are based on CNN. For another reason, our research aims to realize the transfer of model between different users and promote the practical use of HAR. Therefore, when the basic model has been able to achieve a reasonable recognition accuracy (about 90%) for Source users, for the sake of calculation rate, we didn’t use LSTM which needs larger computation or further improve the CNN to achieve higher accuracy on the Source. The parameters of CNN is shown with the introduction of datasets in Section 3.2.

In the work reported in this paper, we studied the transfer learning methods based on features. Three unsupervised methods were chosen. The aim of all three algorithms is to define the differences between the features of source and target, then use a deep learning approach to learn and eliminate these differences. We applied these algorithms to the network depicted in Figure 2. The experiments described here are also valuable for evaluating which method is more suitable for measuring feature variation between different users in an HAR context.

#### 3.1.2. MMD

MMD-based method is a classic algorithm in transfer learning. MMD can be used to judge whether the two distributions *p* and *q* are the same. It is applied as follows:

(1) $$\mathrm{MMD}\left[F,p,q\right]:=sup_{f\in F}\left(E_{p}\left[f\left(x\right)\right]-E_{q}\left[f\left(y\right)\right]\right),$$

If the discrepancy is equal to 0, then the two distributions are considered to be the same. Empirical estimates are typically used to calculate MMD:

(2) $$\mathrm{MMD}\left[F,X,Y\right]=sup_{f\in F}\left(\frac{1}{m}\sum_{i=1}^{m}f\left(x_{i}\right)-\frac{1}{n}\sum_{j=1}^{n}f\left(y_{j}\right)\right),$$

Combining MMD with a deep learning network has led to the development many methods, such as DDC, DAN and others. The basic approach of all of these methods is to extract features from the source and the target datasets using the same network, solve an MMD taking account between features of the two domains at different layers, and use deep learning networks to learn and minimize this discrepancy.

In practical applications, such as DAN, MK-MMD (multiple-kernel MMD) is often used. Multiple kernel functions are used to solve the MMD and calculate the mean of results from every function. Different parameter σ are often set for Gaussian kernel functions to be used as different kernel functions. MK-MMD performs better than MMD. This is proved in DAN [15]. Gaussian kernel function is shown as follow:

(3) $$k\left(x,y\right)=exp\left(\frac{-∥x-y∥{}^{2}}{2\sigma^{2}}\right),$$

#### 3.1.3. DANN

**Figure 3:**
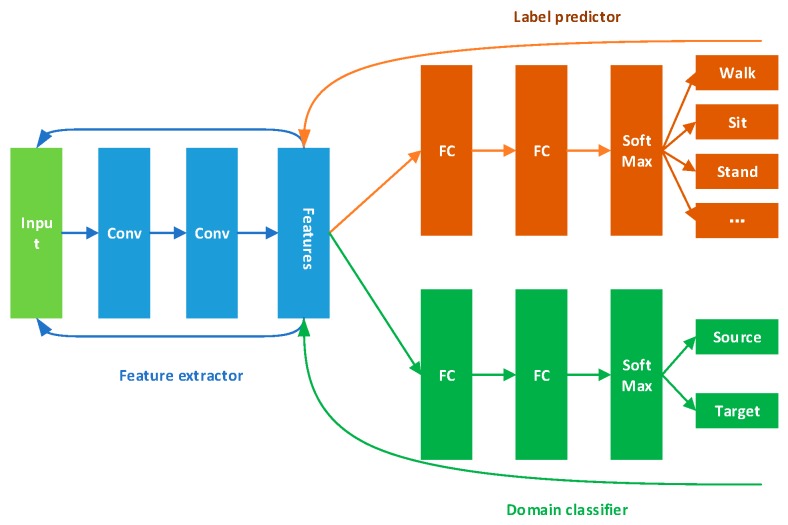
DANN framework, it contains feature extractor, label predictor and domain classifier.

Domain-adversarial Neural Network (DANN) [29] extracts general features through domain confusion between source and target, then it uses these features for classification. Its framework is shown in Figure 3.

The feature extractor in Figure 3 will extract features from both the source and the target data. DANN has two classifiers. One is a label predictor and it is used to predict which category the current data belongs to. The other one is the domain classifier and is used to judge which domain the data comes from. Source data are labeled, and we can use them to train the label predictor. We tag source data with label 0 and target data with label 1 to train the domain classifier,

We want the domain classifier cannot judge whether data comes from source and target. Thus, the feature extractor can extract general features for both two domains. The loss of this classifier should be as large as possible. In the meanwhile, the aim for the label predictor is to make accurate classifications using the general features. We want the loss of this part will be small. One large and one small loss forms the adversarial relationship.

#### 3.1.4. WD

The Wasserstein Distance (WD) is a function for measuring differences between probability distributions in a given metric space (Equation (4)):

(4) W(Pr,Pg)=Eγ∈∏(Pr,Pg)inf(x,y)∼γ[∥x−y∥],

In Equation (4), ∏($P_{r}$, $P_{g}$) represents the set of joint distribution for (x, y). We choose a joint distribution γ from this set and get the expected value of ‖x–y‖ when (x, y) obeys the distribution of γ. The smallest expected value is Wasserstein Distance. One approach [30] has minimized the Wasserstein Distance between source and target in order to facilitate the transfer of the trained model between different domains.

### 3.2. Datasets and Setup

In this work, two data sets are used in the field of activity recognition to conduct experiments: UCI daily and sports dataset [31] and USC-HAD [32]. The information of datasets is shown in Table 1. With the USC-HAD, there are two kinds of activities which are really similar: elevator up and elevator down. We combined them into one activity referring to literature [33], so we get 11 kinds of activity.

For two different datasets, there have some differences in the deep learning network model in Section 3.1.1 (Basic Deep Learning Model). The details are shown in Table 2.

### 3.3. Distribution of Features

Since we want to study the distribution of features, we set two nodes in the first fully connected layers and use the output as 2-dimensional generated features. We randomly select four users (P1, P2, P5 and P7) from the UCI dataset. To analyze clearly, we train and extract features on three activities among them.

**Figure 4:**
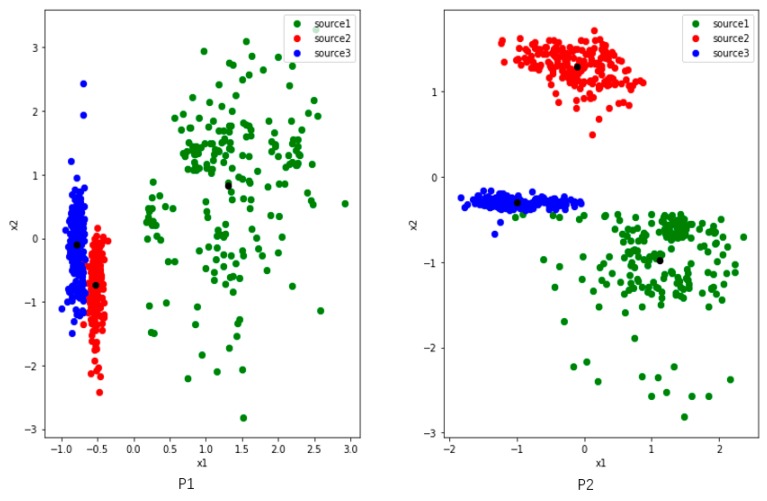
The distribution of three activities features which are collected from users P1 (**left**) and P2 (**right**) in UCI. The red and blue points of P1 are close to each other, while the green one is dispersed and away from them. The blue and green activities of P2 are relatively close, and the green activity is also dispersed. The black points represent the center of features belong to same class. The black points are the centers of the three classes of features.

Figure 4 shows three activities features of P1 and P2, and they have commonalities: (1) there are two colors of points close together. (2) The distribution of green point is comparatively dispersed. We make some definitions here. The features of the user P1 and P2 reflect the problem that the inter-class distance is small while the inner-class distance is large. Of course, this result is also partly limited by the learning ability where the number of nodes is set to 2 in the first fully connected layer.

**Figure 5:**
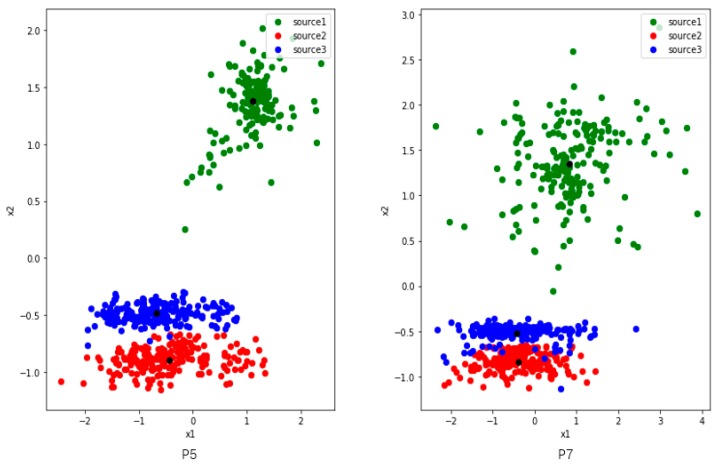
The distribution of three activities features which are collected from users P5 (**left**) and P7 (**right**) in UCI. The distributions of the two users are very similar. The red and blue points are close to each other, while the green one is dispersed and away from them. The black points are the centers of the three classes of features.

Figure 5 shows three activities features of users P5 and P7. The inter-class distance between the blue and red points is small, and the inner-class distance of each class is large, especially the green one. There is a tendency to confuse activities where inter-class distance are too small, such as red and blue. The green features are widely distributed, and they are likely to interfere when new class appears.

We measure and summarize the inter-class distance and the maximum inner-class distance of each class collected from all 4 users in Table 3. We can see that Max_1 is much longer than Distance_1_2 and Distance_1_3 of P1. That is to say, some points in the first class have a larger distance to their corresponding feature center than its inter-class distance. It means if the points are distributed in the area between the feature centers, they are likely to be confused. Max_3 is also much larger than Distance_2_3. As shown in Figure 4, it can be seen that some third activity points is closer to the center of the second activity. Therefore, too small inner-class distance and too large inter-class distance can lead some edge points to get closer to other class, resulting to classification confusion.

A few observations can be drawn:

*Observation 1*: The features of activity data collected by sensors show that the inter-class distance is small. However, the inner-class distance is large.

*Observation 2*: A small distance between classes may lead to the mixing of features. However, a large inner-class distance will make features on the edge more similar to other classes. These two aspects increase the difficulty of recognition.

**Figure 6:**
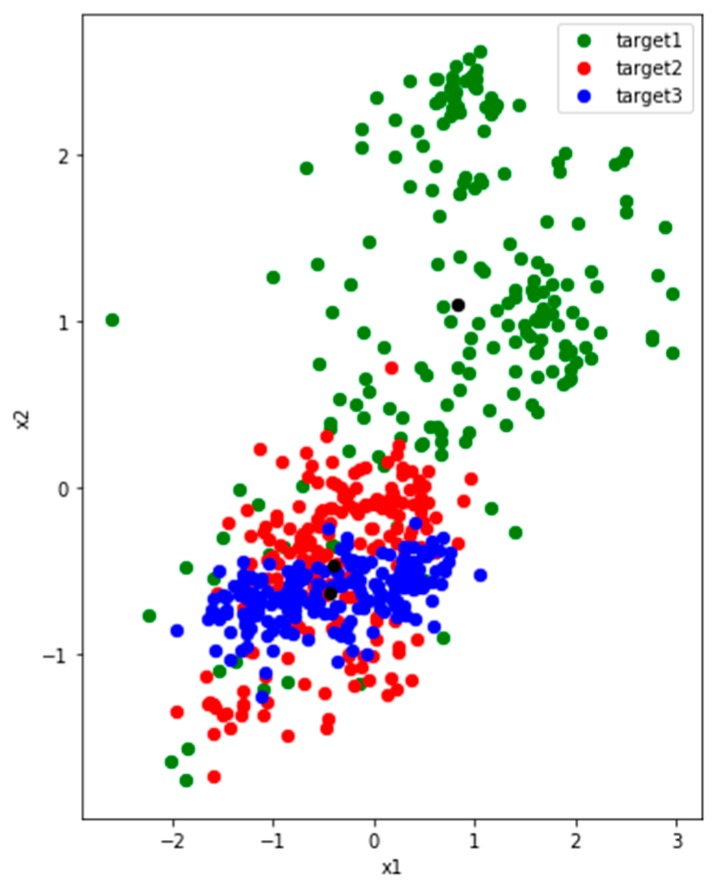
The features for P7 extracted by model trained only with P5 data, which are mixed, dispersed, and indistinguishable. The black points are the centers of the three classes of features.

In Figure 6, we can see that P5 and P7 show similar feature distributions, so we use the P5 data training model to extract features for P7. However, only P5 is learned, the extracting features for P7 are mixed and dispersed as it can be seen in Figure 6. Obviously, it is impossible to classify P7 reasonably and effectively.

*Observation 3*: Due to the difference in data between different users, a model trained for user A may only extract mixed features for user B, which makes prediction of activity difficult.

Our task is to measure the difference between source and target with deep learning framework. Then use deep learning to study and eliminate this difference. We want to make features of target distribute like the ones of source.

### 3.4. Experiments on Existing Methods

In this work, we used a unified network model for different methods, which is exactly the same as Only-Source. The learning rate is 0.0001 and the batch size is 512. The epochs is 20,000 in UCI dataset and 8000 in USC-HAD. In the MMD method, we used MK-MMD (we will simply call it as MMD below), similar to DAN, but only calculate the MK-MMD once after the first fully connected layer.

The purpose of this paper is to transfer between different users. Therefore, our evaluation criteria is the recognition accuracy on Target user data with the model after the transfer learning. In UCI, each user has the same amount of data for each activity, so we used a three-fold cross-validation. To control the variables, for the Source user, we used fixed 2/3 tagged data to participate in the training. In the first round of experiments, the Target object was trained by rotated 2/3 unlabeled data and tested with the remaining 1/3 of the Target. We recorded the best results in 20,000 iterations.

If we use all the users inside the dataset and transfer between them, there are too many arrangements, so we randomly selected some users in UCI which are P1, P2, P5, P7. We transferred between two users each time, one is Source and one is Target. The results are shown in Table 4. In this round of experiments, it was apparent that all the three methods achieved better results than Only-Source. MMD has achieved the best result, and it has positive effect on each user, with the best adaptability. DANN got the worst performance and it didn’t converge sometimes. Since the framework of the three methods are consistent and they measure the discrepancy between source and target in different ways, we believe that MMD is most suitable approach for different users in HAR.

**Figure 7:**
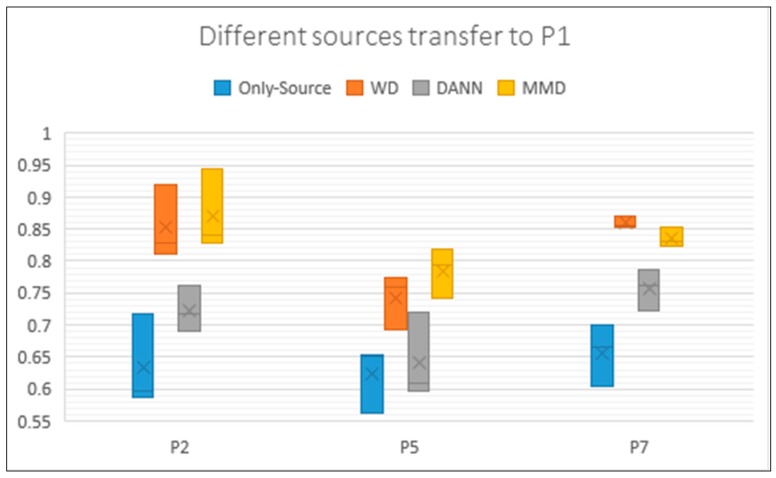
Box plot of the accuracy of P2, P5, and P7 transfer to P1. Each Box feeds back the results of three experiments. It can be seen that some box lines are long and show obvious fluctuations. The y axis represents the accuracy rate of these experiments. The same with the following tables.

Each result in Table 4 is an average of three experiments. To show the details we draw the boxplot (Figure 7). We chose all the transfer experiments of which target is P1. The box in Figure 7 reflects the fluctuation of each method. When using different data of the same Target, the transfer result has a relatively large change and is not stable enough. On the one hand, it is related to the data. On the other hand, we think it is related to parameters.

Because it is difficult to get large amounts of data for target users, In the second round of experiments, we used 1/3 unlabeled data of Target for training and the remaining 2/3 unlabeled data for testing. The acquisition of data in HAR is always a challenging problem.

After target’s unlabeled data is reduced, it is clear that the accuracy of transferring declines which is shown in Table 5. Except DANN, the other two methods still achieve positive transferring, and MMD reached the best result again. DANN performed negative transfer in several pairs and it was not stable. The negative transferring means the result is even worse than not transferring. If there are still methods can work well when the unlabeled data is not large, we think the unsupervised transfer learning has practical value.

We repeated the experiment with another dataset, USC-HAD. Since the amount of data for each person in USC is different, we randomly divided the data into train set and test set. In the first round of experiments, more data was used for target as unlabeled data, accounting for 60%. The rest 40% is used as a test set. The results are shown in Table 6. We can see that MMD has improvement for every pair. WD reached best accuracy in P5~P6, P7~P5 and P9~P5. However, it also had some negative performance in several pairs. DANN also got worse results than only-source in three pairs. Because we want to use the transferring in real life, it needs to be faced with many different users. We think the transfer method do not have to always perform best but it should be stable to work for as more users as possible.

**Figure 8:**
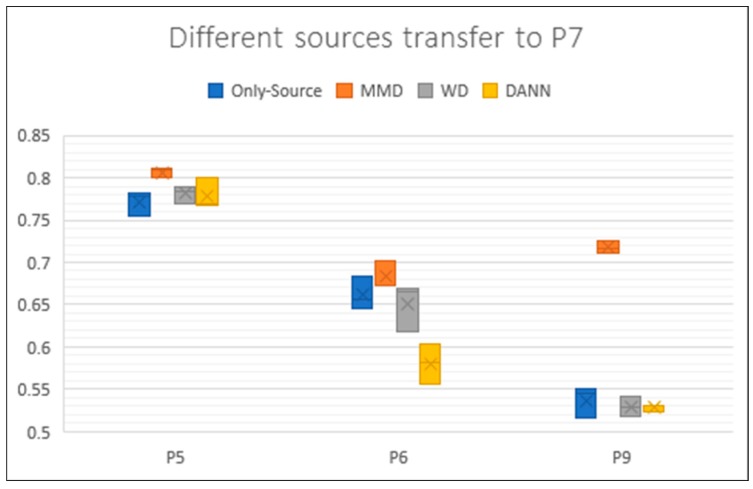
Boxplot of results for P5, P6 and P9 transferring to P7. Each box contains three results. Different colors represent different methods. The y axis represents the accuracy rate of these experiments.

We draw the boxplot for USC as well (Figure 8). We chose all the transfer experiments of which target is P7. Each result is also an average of three experiments. The fluctuation of different data of the same user in USC is smaller than UCI. However, we can still see some changes of accuracy. Using different user as source got very different results which reflects there are really huge differences between activity data of users. This is another problem that the activity data of user will really change during time.

In the second round of experiments, the amount of unlabeled data was reduced to 20%, and the remaining 80% was used as a test set. In Table 7 we can see that MMD still achieved the best accuracy.

Combining the four rounds of results, we can derive the following observations:

*Observation 4*: After transfer learning, better results can be achieved by algorithms than only-source, so it is meaningful to transfer the models between users. The divergence between users truly exists and can be measured by different ways. This is consistent with observation 3.

*Observation 5*: MMD is more effective than the other two methods for measuring the difference between sensor data of different users. It can reach the best result.

*Observation 6*: Small amounts of unlabeled data can generally get positive results in HAR. Therefore, the transferring of models between different users is of practical value.

## 4. Improve Transferring with Adjustment of Features Distribution

We studied the distribution of features for data in HAR and find that inner-class distance is too large and inter-class distance is small. We hope to change this distribution and make classification of source clearer to improve the transferring. We found that MMD performs best during the experiments. We considered using Center Loss [34] in combination with MMD Loss to solve the above problem. 

### 4.1. Center Loss

**Figure 9:**
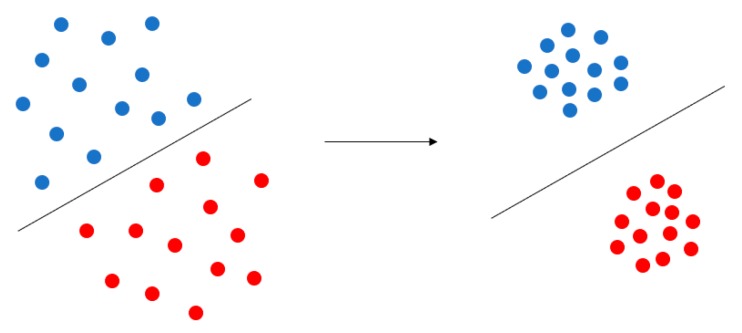
Purpose of Center Loss. Center Loss aggregates the feature of each category to their respective central points, which makes the similar feature more aggregated, and the different types of features spread out, making the classification clearer.

Center Loss was first proposed in the field of face recognition. It is based on the idea that the features be clustered by narrowing the inner-class distance and the features of different classes will spread out. Its purpose is depicted in Figure 9.

Center Loss defines a central point for the features of each class. Then it calculates the distance of each feature from its corresponding central point:

(5) $$L_{c}=\frac{1}{2}\sum_{i=1}^{m}∥\chi_{i}-C_{yi}∥{}^{2}$$

As is shown in Equation (5), m represents batch size, $\chi_{i}$ is the feature of the i-th data, $C_{yi}$ is the corresponding central point. And yi is the label of $\chi_{i}$ which can tell us the class of the central point (C). The total loss is the $L_{s}$ (Soft-max Loss) combined with the $L_{c}$ (Center Loss). λ is used to control their weights.

(6) $$L=L_{s}+\lambda L_{c}$$

The gradient we need to update for Center Loss is $\chi_{i}-C_{yi}$, and we need to update the central points with $\Delta C_{i}$:

(7) $$\frac{\partial L_{c}}{\partial \chi_{i}}=\chi_{i}-C_{yi}$$

(8) ΔCi=∑δi=1m(yi=j)·(Cyi−χi)1+∑δi=1m(yi=j)

### 4.2. Center Loss + MMD (CMMD)

**Figure 10:**
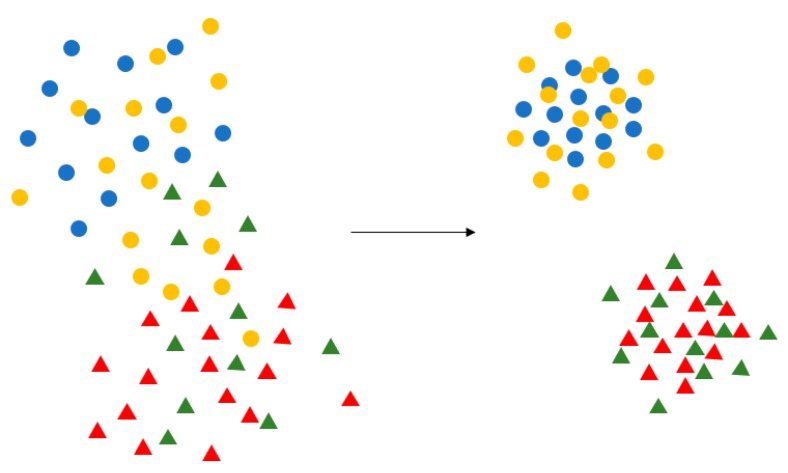
Purpose of Center Loss + MMD.

The algorithm reported in this paper combines Center Loss with MMD Loss. On the one hand, we narrow Center Loss to extract more representative features for Source. On the other hand, we narrow MMD Loss to eliminate the difference between source and target (Figure 10).

We know the expression of MMD in Equation (2). When F is a unit ball in RKHS, MMD can get the best result. In RKHS, we can use dot product of vectors to replace functions f(x):

(9) $$f\left(x\right)={\langle f,\phi (x)\rangle }_{H}$$

ϕ(x) indicates that the original data x is mapped into the Hilbert space (H). All of the functions f for x share this mapping. Since the mapping of x to space H is the same for different f, the final f(x) is determined by the position of f in the space. In RKHS, the dot product of mapping can be calculated by kernel methods:

(10) $${\langle \phi \left(x\right),\phi \left(y\right)\rangle }_{H}=k\left(x,y\right)$$

With the above tools, square the MMD. Since *f* is the point inside the unit ball, a radius that is parallel to $E_{p}\left[\phi \left(x\right)\right]-E_{q}\left[\phi \left(y\right)\right]$ and crosses point *f* can always be found. The calculation process is as shown in Equations (11)–(13), and the kernel function can be used to solve the equation.

(11) $${\mathrm{MMD}}^{2}\left[F,p,q\right]={\left[sup_{∥f\leq 1∥}\left(E_{x\sim p}\left[f\left(x\right)\right]-E_{y\sim q}\left[f\left(y\right)\right]\right)\right]}^{2}$$

(12) $${\mathrm{MMD}}^{2}\left[F,p,q\right]={\left[sup_{∥f\leq 1∥}\langle E_{p}\left[\phi \left(x\right)\right]-E_{q}\left[\phi \left(y\right)\right],f\rangle \right]}^{2}$$

(13) $${\mathrm{MMD}}_{H_{K}}{}^{2}\left[F,p,q\right]={∥E_{p}\left[\phi \left(x\right)\right]-E_{q}\left[\phi \left(y\right)\right]∥}_{H_{K}}{}^{2}$$

H*_K_* represents the kernel function that you used. We used MK-MMD and change the parameter σ of gauss kernel (Expression (3)) to act as different kernels. The MK-MMD Loss is defined as follows:

(14) $$L_{m}=\frac{1}{n}\sum_{k=1}^{n}{\mathrm{MMD}}_{H_{K}}{}^{2}$$

so the total loss is combination of the $L_{s}$ (Soft-max Loss), $L_{c}$(Center Loss) and $L_{D}$(MMD Loss):

(15) $$L=L_{s}+\lambda L_{c}+\beta L_{m}$$

**Figure 11:**
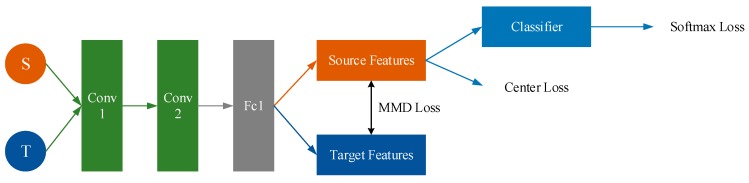
Framework of Center Loss + MMD.

This work uses two layers of CNN and one layer of fully connected layer. When the source and target data are passed through the network, the respective feature of source and target can be obtained. The MMD loss of the current batch can be calculated using source features and target features. The Center Loss can be calculated with source features. The classifier can be trained with source labeled data and we can get the Softmax loss. The framework is shown in Figure 11. The total process of CMMD is shown in Table 8.

### 4.3. Study the Change of Distribution

We studied the changes of feature distribution under Only-Source, MMD, and CMMD. Two kinds of activities are selected from UCI. They are walking in a parking lot (called class 1) and walking on a treadmill with a speed of 4 km/h (called class 2).

**Figure 12:**
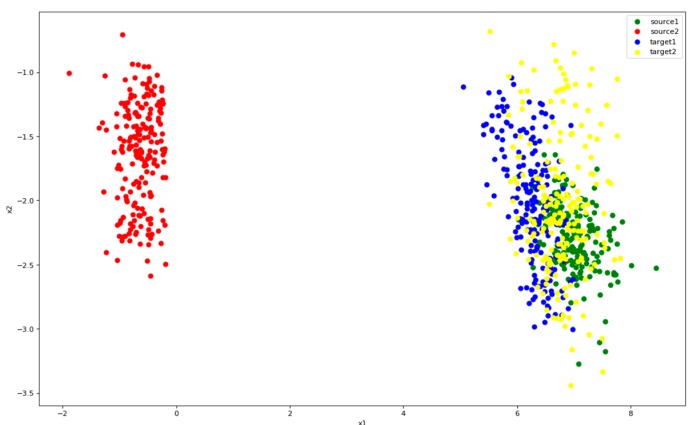
Distribution of features after Only-Source. Color green represents class 1 of source and red represents class 2. Blue is the class 1 of target and yellow the class 2.

Figure 12 shows the distribution of features after the application of Only-Source. It can be seen that the two types of source (green, red) can be clearly separated. But target points (blue, yellow) gather around green points. This pattern indicates that the target’s second category is completely recognized incorrectly. It is necessary to narrow the difference between source and target.

**Figure 13:**
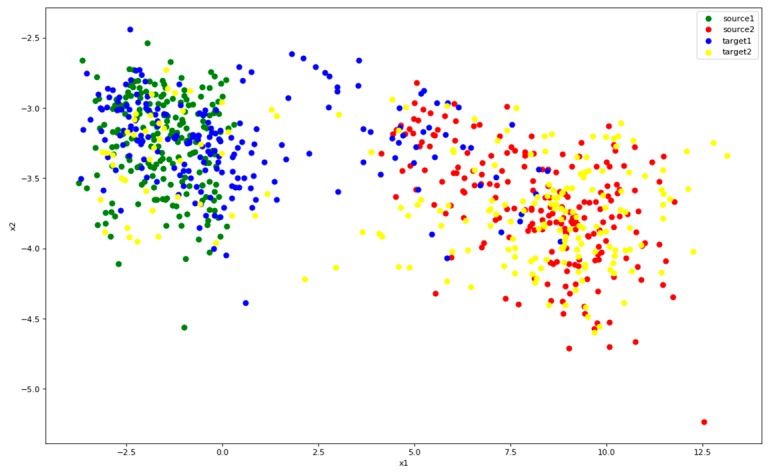
Distribution of features after MMD. Color green represents class 1 of source and red represents class 2. Blue is the class 1 of target and yellow the class 2.

Figure 13 shows the distribution of features after MMD. In this figure, it is apparent that the features of target are defined clearer, and points with the same class from different domains overlap. At this time, the source classifier can accurately recognize a large portion of target data. However, there are still some confusing data which are difficult to recognize, probably because the classification of the source data is not clear enough. MMD can also make features closer to clusters, but it is not sufficient. We will use Center Loss to improve this problem.

**Figure 14:**
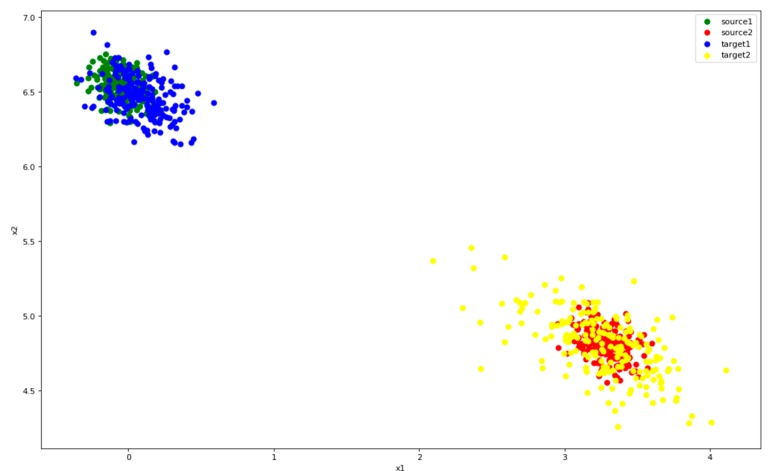
Distribution of features after CMMD. Color green represents class 1 of source and red represents class 2. Blue is the class 1 of target and yellow the class 2.

Figure 14 shows the features after CMMD. By joining center loss, the two types of source features went into clusters. These clusters easy to distinguish and do not impinge upon each other. Therefore, the blue and yellow dots can be better attached to the corresponding colors, even if it is difficult to fit closely, it will not interfere with each other. And spaces can be saved for more classed when features come into clusters.

It is necessary to recognize that these observations are drawn with two-dimensional features. Whether these phenomena still existing with high-dimensional features needs to be proved by experiments. It can be said that the following experiments are used to verify whether these factors exist. This is why we called this work as an empirical study.

### 4.4. Experiments and Evaluation

#### 4.4.1. Experiments on Datasets

In the experiments in Section 3.4, we found that MMD performs best and therefore we use a combination with MMD and Center Loss. From Section 4.3, we can see it works in two-dimensional condition for two kinds of activities. We need experiments to prove decreasing inner-class distance by Center Loss is helpful for transferring in high-dimensional conditions for more activities.

In UCI, the weight of Center Loss was usually set to 1.0E-03 (1.0 × 10^−3^) when the amount of unlabeled data is small, but the actual situation of each transfer task is different, and some needed to be set separately. In Table 9, Large means that more unlabeled data is used, which corresponds to the experimental conditions in Table 4. Small means that fewer unlabeled data is used, which corresponds to Table 5.

It can be seen that under the experiments using two different data levels, the sharpening of the source domain classification is helpful for narrowing the difference between the two domains. CMMD is better than MMD in both data levels. It got better result in 12 pairs and had a higher average when unlabeled data comprised two thirds of the available data. It performed better in 14 pairs and got a better average as well when unlabeled data decline to one third.

In the USC (Table 10), when the weight of Center Loss is set to 1.0E-07 (1.0 × 10^−7^), most of them can achieve better results, different pairs also need some adjustment. It can be seen that after adding Center Loss, the accuracy rate is better than simple MMD. However, from experiments in Section 3 and Section 4, we found that the transfer methods are not stable. We think this may related to parameters and the selection of data. Compared with MMD in many groups of experiments, CMMD does not improve much. The increase of accuracy in some pairs is smaller than 1% and we think these pairs cannot prove the effect of improving feature distribution confidently.

#### 4.4.2. Experiments on Rationality of Improving Feature Distribution

From the result in Section 4.4, we found that CMMD increased obviously by 3.4% in UCI when the amount of unlabeled data from target is small (this is shown in Table 9: MMD-Small and CMMD-Small). We want to repeat this round of experiments and record the results with the value of Center Loss to evaluate whether reducing inner-class distance is helpful.

We analyzed the instability and found that it has a relationship with the initialization of model parameters. Thus, we repeated this part of experiments in UCI with random seed fixed. The other conditions are the same as MMD-Small and CMMD-Small in Table 9. Each pair of users was still transferred to each other three times and take the average. This time we got a relatively stable result (Table 11). There are 36 experiments for each method. CMMD increased by 2.6% in average accuracy.

When using different part of unlabeled data from target, the results still have changes, but the change is more stable when random seed is fixed. When user engage in an activity for a long time, the body movements do change with strength declined and environment changes.

We recorded the final Center Loss value for each experiment. Center Loss can be easily understood as the sum of the inner-class distance of each point in every classes, so it reflects the change of the inner-class distance. In Table 12, we can see that the value of Center Loss has become small after MMD, which we mentioned in Section 4.3. After CMMD, Center Loss has become smaller. Combined with the results in Table 11, we believe that reducing the inner-class distance by Center Loss can help the transfer. The relationship between transfer learning methods and hyperparameters deserves in-depth study.

In the above, we suspected the observations in two-dimensional features exist for high-dimensional ones. Now we can prove it by these experiments. When the Center Loss is smaller, the inner-class distance is smaller, and the accuracy improved. From the results, we can see that there is indeed room for improvement on inner-class and inter-class distances. The phenomena watched on two-dimensional features truly exist on high-dimensional ones.

*Observation 7:* The addition of Center Loss is helpful for MMD. Reducing inner-class distances which can make the features of source more representative and make its classification clearer, contributes to transferring to targets.

## 5. Insights

In this paper, we made some observations, reached some conclusions about them and obtained some insights, listed here:

*Insight 1*: For the human activity data collected by sensors in these two datasets, the inter-class distance is small, but the inner-class distance is large. These factors make features on the edge closer to other classes which make it difficult to recognize and limit the effect of transferring.

*Insight 2*: Due to the differences between users, the models trained for existing users often extract scattered and mixed features for new users, which causes great difficulties for classification. Therefore, the transferring of the model is necessary.

*Insight 3*: Unsupervised Transfer Learning can work well between different people. In UCI, using these existing transfer methods has an average improvement of 13.2% over the Only-Source on accuracy rate and about 4.6% in USC. When the amount of unlabeled data from target is small, it still works. Considering that a large amount of data is difficult to get in a short time, the unsupervised transferring of the model between users is practical. The selection of source user has a huge impact on results.

*Insight 4*: Under the same model conditions, we found that MMD method can achieve the best and it has a positive performance for each user. Therefore, we believe that the MMD is most suitable for measuring the discrepancy between different users in HAR.

*Insight 5*: The principle of Center Loss is to reduce the distance of each feature to the center point of its corresponding category. When the inner-class distance is smaller, the different classes will naturally separate from each other.

*Insight 6*: Combining Center Loss with MMD improves the accuracy of MMD by an average of 1.8%. On the one hand, it reduces inner-class distance by reducing Center Loss to extract more representative features for source. On the other hand, it narrows the differences between source and target by reducing MMD Loss.

## 6. Conclusions and Outlooks

In this work, we focused on the transfer of the deep learning model between users with targets only having unlabeled data. We hope this work will contribute to making HAR closer to our lives. This paper conducts empirical research analysis. We conducted an experimental comparison of three existing unsupervised transfer methods and found that MMD works best. We analyzed the features distribution and found that the inner-class distance is too far, and the inter-class distance is too small. It is speculated that improving such a distribution pattern can help transfer. We used Center Loss to reduce the inter-class distance and transfer with MMD, the effect is improved compared to the simple MMD. This proves that there is indeed room for improving feature distribution in the transferring for HAR.

However, we also need to realize that these experiments are carried out inside the data set. The data sets are collected for users with similar age and posture. These data are far from the actual life situation. In actual situations, the differences between different people will be even greater. In order to make the algorithm work well for any object, there are still many problems that need to be solved. Firstly, when collecting data for users in real life, it will come in a time series. It is impossible to get enough unlabeled data at one time. Online learning needs to be combined to make the transfer more effective. Secondly, people may seldom perform some kinds of activities like running very fast and falling down. Some classes will be absent when the data come. How will transfer learning perform when some classes of data are missed? This is also an important question. Last but not least, the reduction of computation plays an essential role when we want to apply the models on mobile devices. We will focus on these problems in our follow-up work.

## Figures and Tables

**Table 1:** Information of the datasets we used. The link of them is on the name.

Name	Sensors	Users	Activities
UCI daily and sports	A G M ^1^	8	19
USC-HAD	A G	14	12

^1^ A: accelerometer. G: gyro. M: magnetometer.

**Table 2:** Neural network parameters on different datasets. We only make convolution calculation on rows and keep the information of columns.

Parameter	UCI	USC
Number of convolution kernels	50	50
Size of convolution kernels	5 × 1	5 × 1
Size of pooling kernels	3 × 1	3 × 1
Number of neurons in FC layer	1024	500

**Table 3:** The inter-class distance and the maximum inner-class distance of each class collected from all 4 users. Distance_a_b represents the inter-class distance between class a and class b. Max_a represents the maximum inner-class distance of class a.

Distance	P1	P2	P5	P7
Distance_1_2	2.4066	2.5843	2.7499	2.4965
Distance_1_3	2.2896	2.2208	2.5856	2.2583
Distance_2_3	0.6839	1.8230	0.4716	0.3203
Max_1	3.6419	2.1996	1.6846	3.2121
Max_2	1.1788	1.0435	1.1338	1.2189
Max_3	1.5911	0.9664	1.2174	1.2210

**Table 4:** The first round of experiment results of UCI. In this table, we show the accuracy of four methods for Target. P1 is Source, P2 is Target, and P1~P2 means transfer from P1 to P2. Because of the cross-validation used, each data in the table is the mean value of three experiments. The bold number is the best result for the experiments in the same row.

Pairs	Only-Source	MMD	DANN	WD
P1~P2	0.6037	**0.9367**	0.7381	0.8611
P2~P1	0.6340	**0.8711**	0.7230	0.8525
P1~P5	0.6154	**0.9265**	0.7781	0.9247
P5~P1	0.6228	**0.7847**	0.6421	0.7421
P1~P7	0.6877	**0.9019**	0.8205	0.8656
P7~P1	0.6570	0.8356	0.7565	**0.8596**
P2~P5	0.6916	**0.8925**	0.8218	0.7988
P5~P2	0.6100	**0.8516**	0.7016	0.8374
P2~P7	0.7440	0.9507	0.8161	**0.9598**
P7~P2	0.6839	0.9163	0.7086	**0.9253**
P5~P7	0.5397	0.7788	0.6453	**0.8188**
P7~P5	0.5637	0.7982	0.7030	**0.8100**
Average	0.6378	**0.8704**	0.7379	0.8546

**Table 5:** The second round of experiment results of UCI. In this round of experiments, we reduced the amount of Target unlabeled data for training transfer to 1/3 and increased the test set to 2/3. The bold number is the best result for the experiments in the same row.

Pairs	Only-Source	MMD	DANN	WD
P1~P2	0.7226	**0.8527**	0.7209	0.8112
P2~P1	0.7340	**0.8241**	0.6671	0.8135
P1~P5	0.6451	**0.9141**	0.6591	0.8968
P5~P1	0.6375	**0.7667**	0.6615	0.7425
P1~P7	0.7710	0.8608	0.7625	**0.8660**
P7~P1	0.7254	0.8425	0.7255	**0.8593**
P2~P5	0.7797	**0.8930**	0.7559	0.8413
P5~P2	0.7074	**0.8275**	0.6823	0.8088
P2~P7	0.8079	**0.9542**	0.7974	0.9483
P7~P2	0.7199	0.9099	0.7127	**0.9186**
P5~P7	0.5857	0.7364	0.6254	**0.7847**
P7~P5	0.7220	**0.8411**	0.6674	0.8346
Average	0.7132	**0.8519**	0.7031	0.8438

**Table 6:** The first round of experiment results of USC. We also randomly selected 4 users from the USC, namely P5, P6, P7 and P8, and transferred between each pair. In this round of experiments, Target used 60% of the unlabeled data to participate in the training, using the remaining 40% for testing. The bold number is the best result for the experiments in the same row.

Pairs	Only-Source	MMD	DANN	WD
P5~P6	0.5965	**0.6232**	0.6205	0.5775
P6~P5	0.5639	0.5855	0.5318	**0.6084**
P5~P7	0.7713	**0.8063**	0.7788	0.7814
P7~P5	0.6400	0.7099	0.6912	**0.7292**
P5~P9	0.5543	**0.6627**	0.5429	0.5331
P9~P5	0.4874	**0.6787**	0.5224	0.5744
P6~P7	0.6625	**0.6828**	0.5802	0.6507
P7~P6	0.5342	0.5641	0.5580	**0.6434**
P6~P9	0.3492	**0.4483**	0.4145	0.2995
P9~P6	0.4370	**0.6640**	0.5495	0.4818
P7~P9	0.4244	**0.5808**	0.4994	0.4485
P9~P7	0.5370	**0.7174**	0.5286	0.5293
Average	0.5465	**0.6436**	0.5682	0.5714

**Table 7:** The second round of experiment results of USC. In this round of experiments, Target only used 20% of the unlabeled data to participate in the training, using the remaining 80% for testing. The bold number is the best result for the experiments in the same row.

Pairs	Only-Source	MMD	DANN	WD
P5~P6	0.6001	**0.6152**	0.6074	0.5925
P6~P5	0.5698	0.5761	0.5280	**0.6109**
P5~P7	0.7694	**0.8063**	0.7754	0.7735
P7~P5	0.6472	0.7217	0.6873	**0.7351**
P5~P9	0.5549	**0.6561**	0.5405	0.5965
P9~P5	0.4825	**0.6793**	0.4792	0.6235
P6~P7	0.6591	**0.6753**	0.5691	0.6596
P7~P6	0.5245	0.5645	0.5323	**0.6165**
P6~P9	0.3469	**0.4431**	0.4173	0.3001
P9~P6	0.4442	**0.6665**	0.5102	0.4751
P7~P9	0.4387	**0.6658**	0.4963	0.4401
P9~P7	0.5436	**0.7235**	0.5256	0.4931
Average	0.5484	**0.6495**	0.5557	0.5764

**Table 8:** The flow of the CMMD algorithm.

Center Loss with MMD
Input: Training Data [$X_{Source},\;X_{Target}$] Label $Y_{Source}$
Hyperparameter λ,α,β, learning rate $\mu^{t}$, epochs t
Output: Neural network parameter$\;\theta_{c}$
1: while not converge do 2: t←t+1
3: compute total loss: $L^{t}=L_{s}^{t}$ + $L_{C}^{t}$ + $L_{m}^{t}$ 4: compute gradient: $\frac{\partial L^{t}}{\partial X_{i}^{t}}$ = $\frac{\partial L_{S}^{t}}{\partial X_{i}^{t}}$ + $\lambda \cdot \frac{\partial L_{C}^{t}}{\partial X_{i}^{t}}$ + $\beta \cdot \frac{\partial L_{m}^{t}}{\partial X_{i}^{t}}$
5: update central points: $C_{j}^{t+1}=C_{j}^{t}-\alpha \cdot \Delta C_{j}^{t+1}$ 6: update network parameter: $\theta_{C}^{t+1}=\theta_{C}^{t}-\mu^{t}\sum_{i}^{m}\frac{\partial L^{t}}{\partial X_{i}^{t}}\cdot \frac{\partial X_{i}^{t}}{\partial \theta_{C}^{t}}$. 7: end while

**Table 9:** Experiment result of MMD and CMMD on UCI. Large indicates that the Target uses more unlabeled data under the experimental conditions shown in Table 4. Small indicates that the Target uses less unlabeled data under the experimental conditions shown in Table 5. The bold result means it is bigger than another result in the same row with the same condition (Large or Small). The same with Table 10.

Pairs	MMD-Large	CMMD-Large	MMD-Small	CMMD-Small
P1~P2	**0.9367**	0.9289	0.8527	**0.9061**
P2~P1	0.8711	**0.8781**	0.8241	**0.8344**
P1~P5	0.9265	**0.9625**	0.9141	**0.9634**
P5~P1	0.7847	**0.7961**	0.7667	**0.7954**
P1~P7	**0.9019**	0.8788	0.8608	**0.8873**
P7~P1	0.8356	**0.8449**	**0.8425**	0.8403
P2~P5	0.8925	**0.9209**	0.8930	**0.9725**
P5~P2	0.8516	**0.8570**	**0.8275**	0.8132
P2~P7	**0.9507**	0.9437	**0.9542**	0.9525
P7~P2	0.9163	**0.9421**	0.9099	**0.9325**
P5~P7	0.7788	**0.8589**	0.7364	**0.8469**
P7~P5	0.7982	**0.8586**	0.8411	**0.8890**
Average	0.8704	**0.8892**	0.8519	**0.8861**

**Table 10:** Experiment result of CMMD on USC. Large indicates that the Target uses more unlabeled data under the experimental conditions shown in Table 6. Small indicates that the Target uses less unlabeled data under the experimental conditions shown in Table 7.

Pairs	MMD-Large	CMMD-Large	MMD-Small	CMMD-Small
P5~P6	0.6232	**0.6242**	0.6152	**0.6181**
P6~P5	0.5855	**0.5871**	0.5761	**0.5958**
P5~P7	0.8063	**0.8089**	0.8063	**0.8111**
P7~P5	0.7099	**0.7597**	0.7217	**0.7614**
P5~P9	**0.6627**	0.6409	**0.6561**	0.6329
P9~P5	0.6787	**0.6849**	**0.6793**	0.6575
P6~P7	**0.6828**	0.6772	**0.6753**	0.6750
P7~P6	0.5641	**0.6020**	0.5645	**0.5984**
P6~P9	0.4483	**0.4922**	0.4431	**0.4938**
P9~P6	0.6640	**0.6700**	0.6665	**0.6718**
P7~P9	0.5808	**0.5992**	0.6658	**0.6728**
P9~P7	0.7174	**0.7216**	0.7235	**0.7303**
Average	0.6436	**0.6557**	0.6495	**0.6599**

**Table 11:** The experiments on UCI with the fixed random seed for initialization and the other conditions unchanged as MMD-Small and CMMD-small in Table 9. The bold number in this table means the biggest average in the same row for the two methods with three experiments.

Pairs	MMD				CMMD			
	Expt1	Expt2	Expt3	Average	Expt1	Expt2	Expt3	Average
P1~P2	0.8316	0.9289	0.9347	0.8984	0.8476	0.9476	0.9329	**0.9094**
P2~P1	0.8032	0.8571	0.8482	**0.8361**	0.8134	0.8374	0.8405	0.8304
P1~P5	0.8550	0.8745	0.9318	0.8871	0.9471	0.9674	0.9568	**0.9571**
P5~P1	0.7861	0.7616	0.7482	0.7653	0.7597	0.8216	0.7953	**0.7922**
P1~P7	0.8424	0.8587	0.8721	0.8577	0.8613	0.8779	0.8600	**0.8664**
P7~P1	0.8276	0.8397	0.8437	**0.8370**	0.8105	0.8482	0.8182	0.8256
P2~P5	0.8145	0.8276	0.8187	0.8203	0.9726	0.9889	0.9589	**0.9735**
P5~P2	0.8195	0.8979	0.8584	**0.8586**	0.7942	0.8089	0.7679	0.7904
P2~P7	0.9100	0.9453	0.9692	**0.9415**	0.9129	0.9355	0.9476	0.9320
P7~P2	0.9168	0.9350	0.8721	0.9080	0.8987	0.9539	0.9029	**0.9185**
P5~P7	0.7718	0.7266	0.7468	0.7484	0.8379	0.7732	0.7679	**0.7930**
P7~P5	0.7637	0.7679	0.7766	0.7694	0.8634	0.8724	0.8350	**0.8569**
Average				0.8440				**0.8705**

**Table 12:** The final Center Loss value in each experiment.

Pairs	Center Loss in MMD				Center Loss in CMMD			
	Expt1	Expt2	Expt3	Average	Expt1	Expt2	Expt3	Average
P1~P2	7.0641	6.0552	5.8829	6.3341	2.5095	3.9016	4.3656	3.5923
P2~P1	4.3023	4.0079	3.9652	4.0918	3.3859	2.4400	2.1673	2.6644
P1~P5	5.6797	5.7204	6.2997	5.8999	2.0288	2.6139	2.1705	2.2711
P5~P1	3.0684	3.1343	2.8156	3.0061	1.3397	1.5934	1.3735	1.4356
P1~P7	5.8804	4.4947	6.2086	5.5279	2.3206	2.7495	2.3340	2.4680
P7~P1	3.0229	3.0714	2.8922	2.9955	1.3355	1.2104	1.2586	1.2682
P2~P5	3.7914	3.7328	3.8657	3.7966	1.5664	1.3864	1.6750	1.5426
P5~P2	3.2929	4.1160	3.8489	3.7526	1.3598	1.4336	0.5780	1.1238
P2~P7	4.1270	3.6414	3.3205	3.6963	1.6238	1.4944	1.4380	1.5187
P7~P2	2.7896	3.5230	3.0606	3.1244	1.2902	1.2553	1.2591	1.2682
P5~P7	3.6943	3.9466	4.1478	3.9296	1.3651	1.2760	1.4209	1.3540
P7~P5	3.7604	3.6678	3.7770	3.7351	1.4640	1.4026	1.4796	1.4487
